# Acute Heat-Evoked Temperature Sensation Is Impaired but Not Abolished in Mice Lacking TRPV1 and TRPV3 Channels

**DOI:** 10.1371/journal.pone.0099828

**Published:** 2014-06-12

**Authors:** Irène Marics, Pascale Malapert, Ana Reynders, Stéphane Gaillard, Aziz Moqrich

**Affiliations:** Aix-Marseille-Université, CNRS, Institut de Biologie du Développement de Marseille, UMR 7288, Marseille, France; St. Joseph's Hospital and Medical Center, United States of America

## Abstract

The discovery of heat-sensitive Transient Receptor Potential Vanilloid ion channels (ThermoTRPVs) greatly advanced our molecular understanding of acute and injury-evoked heat temperature sensation. ThermoTRPV channels are activated by partially overlapping temperatures ranging from warm to supra-threshold noxious heat. TRPV1 is activated by noxious heat temperature whereas TRPV3 can be activated by warm as well as noxious heat temperatures. Loss-of-function studies in single TRPV1 and TRPV3 knock-out mice have shown that heat temperature sensation is not completely abolished suggesting functional redundancies among these two channels and highlighting the need of a detailed analysis of TRPV1::TRPV3 double knock-out mice (V1V3dKO) which is hampered by the close proximity of the loci expressing the two channels. Here we describe the generation of a novel mouse model in which *trpv1* and *trpv3* genes have been inactivated using bacterial artificial chromosome (BAC)-based homologous recombination in embryonic stem cells. In these mice, using classical thermosensory tests such hot plate, tail flick and the thermotaxis gradient paradigms, we confirm that TRPV1 is the master channel for sensing noxious heat temperatures and identify a cooperative role of TRPV1 and TRPV3 for sensing a well-defined window of acute moderate heat temperature. Using the dynamic hot plate assay, we unravel an intriguing and unexpected pronounced escape behavior in TRPV1 knock-out mice that was attenuated in the V1V3dKO. Together, and in agreement with the temperature activation overlap between TRPV1 and TRPV3 channels, our data provide *in vivo* evidence of a cooperative role between skin-derived TRPV3 and primary sensory neurons-enriched TRPV1 in modulation of moderate and noxious heat temperature sensation and suggest that other mechanisms are required for heat temperature sensation.

## Introduction

Sensing environmental and internal temperature is a key factor that allows individuals to maintain their thermal homeostasis and to avoid tissue-damaging noxious temperatures. Temperature sensation has long been suggested to be primarily mediated by specialized sensory neurons whose cell bodies located in trigeminal and dorsal root ganglia (DRG). A breakthrough in our molecular understanding of thermosensation occured when Caterina and colleagues identified the heat and capsaicin receptor TRPV1 [1]. TRPV1 belongs to a large superfamily of Transient Receptor Potential ion channels that can be subdivided into six main subfamilies: the TRPC (canonical), TRPM (melastatin), TRPP (polycystin), TRPML (mucolipin), the TRPA (ankyrin) and TRPV (vanilloid) groups [2]–[5]. The TRPV family contains six members among which TRPV4, TRPV3, TRPV1 and TRPV2, dubbed ThermoTRPVs, are activated by partially overlapping temperatures ranging from warm to supra-threshold noxious heat [1], [3], [6]–[10]. Although activated at specific temperature thresholds, loss-of-function studies in mice have shown that thermosensation is not completely abolished, suggesting some functional redundancy among these channels [11]–[15]. For example, acute noxious heat sensitivity is impaired but not completely abolished in TRPV1-KO mice as well as in transgenic mice constitutively expressing the short hairpin RNAs that silence the ***Trpv1*** gene [12], [15], [16], suggesting that other receptors in concert with TRPV1 may contribute to moderately noxious heat temperature sensitivity. Amongst thermo-TRPV channels, the three potential candidates that could compensate for loss of TRPV1 are TRPV2, TRPV3 and TRPV4. TRPV2 has an activation threshold around 52°C in vitro [10], but mice lacking TRPV2 show normal behavioral responses to noxious heat over a broad range of temperatures [13]. TRPV3, which is highly enriched in rodent keratinocytes, has an activation threshold around 33°C, and its activity increases at noxious temperatures *in vitro* [6], [7], [17]. TRPV3-KO mice exhibit a defect in warm temperature sensation but have subtle deficit in response to acute noxious heat stimuli [11]. A recent study has shown that mice deficient in TRPV3 and TRPV4 in a C57BL/6J background exhibit no alteration in warm or noxious heat temperature perception [18]. Furthermore, mice deficient in both TRPV1 and TRPV2 have no additive phenotype in response to acute noxious heat stimuli [13], suggesting that moderate noxious heat temperature sensation is mainly mediated by TRPV1 channel but opening the possibility for TRPV3 to be the likely thermoTRPV candidate that contributes to the residual heat sensitivity encountered in TRPV1 knockout mice [1], [15].

In this study we evaluated the extent to which these two channels can functionally overlap *in vivo* by generating TRPV1 and TRPV3 double knock-out mice. In these mice, using a series of thermosensory tests, including hot plate, tail flick, thermotaxis gradient and dynamic hot plate, we confirmed the predominant role of TRPV1 in perception of noxious heat temperature sensation and demonstrated that TRPV1 and TRPV3 channels cooperate with each other to sense a well-defined window of acute moderate heat temperature. Our data also highlight the likely involvement of other molecular players in heat temperature sensation.

## Materials and Methods

### Animals

All protocols were in agreement with European Union recommendations for animal experimentation and approved by scientific and ethics committees (agreement 14-08112010- Marseilles ethics committee). In addition the protocol for disruption of *Trpv1* and *Trpv3* genes has been approved by the institutional committee at the Villejuif facility GMO number 5847 for generation of ES cells and number 2009/4857 for housing genetically modified mice models. TRPV1 and TRPV3 knock-out mice were purchased from the Jackson Laboratory. Mice were maintained under standard housing conditions (23°C, 40% humidity, 12 h light/dark cycles, and free access to food and water). Special effort was made to minimize the number as well as the stress and suffering of mice used in this study.

### Generation of TRPV1-TRPV3 double KO (V1V3dKO) mice

#### Targeting vector

The targeting vector was constructed on the basis of a 205 kb genomic clone containing both *Trpv1* and *Trpv3* genes (BAC RP23-388F9), obtained from a 129SVJ “BACPAC” Resources Center (BPRC) library. Both 4.5 kb *Trpv1*-5′arm and 300 bp *Trpv3*-3′arm were chosen in order to delete a 18.9 kb region starting at exon 11 of *Trpv1* up to the end of exon 2 of *Trpv3*. These homology arms were amplified from BAC RP23-388F9 by PCR using Phusion High-Fidelity DNA polymerase (Finnzymes). A Venus-SV40 polyA cassette was fused in frame with the *Trpv1*-5′ arm, and a LoxP-flanked PGK-EM7-Neo selection cassette was introduced downstream of the Venus SV40 polyA and upstream of the *Trpv3*-3′ arm.

#### Targeted disruption of murine *Trpv1 and Trpv3* genes

The targeting cassette was electroporated in RP23-388F9-containing DH10B bacteria and clones were screened for homologous recombination. At all steps, BAC integrity and digestion were verified by pulsed field gel electrophoresis (CHEF-DR II system, Bio-Rad). The 5′arm of homology recombinant extends over 4.5 kb and the 3′arm over 150 kb. Electroporation of the linearized recombinant BAC was performed into 129sv-derived embryonic stem cells (CK35) at the CNRS-SEAT transgenic facility (Villejuif, France). Recombinant ES clones were identified by southern blot using a 5′ external probe and a neo probe. Two different targeted clones were injected into C57BL/6J-derived blastocysts at the CNRS-SEAT transgenic facility (Villejuif, France). Resulting chimeras were mated to C57BL/6J females to produce germ-line transmission of the recombinant allele. The following oligonucleotides were used for offspring genotyping: 5′F (TTTTGCTTCCCAACATGTCA), 3′R1 (GTTACCTGTGGAAAATCCAAACAAGAAC) and 3′R2 (CAGATCAGCTTCAGGGTCAGCTTG).

#### Detection of *Trpv1* and *Trpv3* transcripts

DRG and hairy back skin were dissected from adult mice in RNA*later* (Qiagen) then put in TRIzol reagent (Life Technologies-Invitrogen). Tissues were dissociated using the Precellys24 homogenizer (Ozyme) and RNA extracted according to the TRIzol reagent protocol, followed by a LiCl precipitation. cDNAs were synthetized by RT-PCR using a mix of oligodT15 and random hexamers as primers and ImPromII reverse transcriptase (Promega). Reverse transcription was carried out for 1 h at 42°C. PCR were performed on the different cDNAs using TRPV1 (V1-F: CTTCTTCCGAGGGATCCAGTATT); V1-R: CCTCATGCACTTCAGGAAACTCT) and TRPV3 (V3-F: AGGCTTCTATTTTGGCGAGACAC; V3-R: TCCCGAGGACGGTAGTAAGAGAC) primers.

Detection of TRPV1 protein: to obtain adult tissues, mice were deeply anesthetized using a mix of ketamine/xylazin and then transcardially perfused with an ice-cold solution of paraformaldehyde (PFA) 2% in PBS. After dissection, tissues were post-fixed in the same fixative for 3 hours at 4°C. Tissues were then transferred into 30% sucrose solution for 24 hours and embedded in OCT prior to storage at −80°C. Samples were sectioned (12 µm) using a standard cryostat (Leica). Immunofluorescence was performed following standard protocols, and the primary antibodies used were: TRPV1 antibody (1/1000 dilution, Neuromics), CGRP antibody (1/1000 dilution, Calbiochem), TrkA antibody (1/1000 dilution, Abgent), PGP9.5 antibody (1/1000 dilution, Thermo Scientific), IB4 –A488 (1/200 dilution, Invitrogen).

### Behavioral assays

All behavior analyses were conducted on littermate males 9–15 weeks old. Animals were acclimated for one hour to their testing environment prior to all experiments that were done at room temperature. Experimenters were blind to the genotype of the mice during testing. Gradient, thermal plates, Hargreaves and Von Frey apparatus were from Bioseb.

Open field: Mice were placed individually in a square arena (40×40×35 cm). Locomotor activity was recorded for 5 minutes using Bioseb tracking software. The time spent in the center area versus the corners of the arena is recorded.

Hot plate: To assess heat sensitivity, mice were individually confined in a 20 cm high Plexiglas cylinder on a metal surface set at 46°, 48°, 50°, 52° or 55°C and the latency to nociceptive response (licking, shaking of hind paws or jumping) measured. To prevent tissue damage, mice were removed from the plate immediately after a nociceptive response or a cut-off time of 300 s, 90 s, 60 s, 45 s or 30 s respectively. Each mouse was tested three times with a latency of at least 5 min between each test. The withdrawal time corresponds to the mean of the three measures. The mice were tested for one temperature per day in order to avoid habituation.

Tail immersion: Mice were immobilized with a tissue and the tip of the tail (1/3 of the length) was immersed in a water bath maintained at 46°, 48°, 50° or 52°C. The latency to nociceptive response (withdrawal of the tail) was measured. To prevent tissue damage, the tail was removed from the bath immediately after a nociceptive response or a cut-off time of 60 s, 30 s, 20 s or 20 s respectively. Each mouse was tested three times with a minimum latency of 5 min between each test. The withdrawal time corresponds to the mean of the three measures. The mice were tested for one temperature per day in order to avoid habituation.

Hargreaves' test (thermal nociceptive threshold): To assess hind paw heat sensitivity, Hargreaves' test was conducted using a plantar test device. Mice were placed individually into Plexiglas chambers on an elevated glass platform and allowed to habituate for at least 30 minutes before testing. A mobile radiant heat source of constant intensity was then applied to the glabrous surface of the paw through the glass plate and the latency to paw withdrawal measured. Paw withdrawal latency is reported as the mean of three measurements for both hind paws with at least a 5 minutes pause between measurements. IR source was adjusted to 25% and a cut-off of 20 s was applied to avoid tissue damage. Mice were first challenged to measure the baseline (acute response). After a 10 µl injection of Complete Freund's Adjuvant (Sigma) into the left hind paw, thermal hyperalgesia was determined on day 1, day 3 and day 7 post-CFA.

Up-and-down Von Frey: Animals were individually placed in Plexiglas chambers with a wire mesh floor, and allowed to habituate for at least 2 hours prior to testing. Mechanical responses were tested by stimulating the middle plantar surface of both hind paws with von Frey filaments using a modified “up-and -down method” starting with 1 g then ranging from 0.07 g to 2 g. Biting, licking and withdrawal during or immediately following the 3 s stimulus were considered as a positive response. The strength of the Von Frey filament was increased or decreased following a negative or positive response respectively. This up-down procedure was applied 4 times following the first change in response and stimuli were not re-applied within a 5 minutes period. The 50% response probability for each paw was calculated using the following formula: 50% g threshold  =  (10^[xf+κδ]^)/10.000, where X_f_  =  value of the final filament (in log units), K  =  tabular value, δ  =  mean difference between stimuli (in log units). Data are presented as paw withdrawal threshold (in g) for each group ± SEM.

Mice were tested at D0, prior to a 10 µl injection of Complete Freund's Adjuvant (CFA- Sigma) into the left hind paw, and were tested again on day 1, day 3 and day 7 post-CFA.

Thermal gradient test: temperature gradient assay was performed as described previously (Moqrich et al., 2005). Briefly, mice were individually tracked for 90 min in two separate arenas of the thermal gradient apparatus. A controlled and stable temperature gradient from 14°C to 55°C was maintained using two Peltier heating/cooling devices positioned at each end of the aluminium floor. Each arena was virtually divided into 15 zones of equal size (8 cm) with a distinct and stable temperature. The temperature at the borders of each zone was weekly recorded using an infrared thermometer (testo831- VWR). The tracking was performed using a video camera controlled by the software provided by the manufacturer and the time spent in each zone over the 90 min period determined.

Dynamic Hot Plate (DHP): Mice were individually confined in an 11.5 cm by 11.5 cm by 20 cm Plexiglas arena, on a metal surface set at 34°C. As soon as the mice were put on the surface, a linear gradient of temperature was applied, increasing by 1°C per minute during 10 min, reaching 44°C at the end of the test. The number of jumps (escape behavior) during each 1°C-interval was scored and the temperature of the first jump noted. Animals were habituated for 10 min at 24°C the day before experiment. No vocalization was ever observed during the tests and the arena was high enough so the mice cannot reach the lid.

### Statistical analysis

Data are expressed as mean ± SEM. Data from behavioral experiments were analyzed using Student t tests for experiments with planned comparison designs. Statistical analyses were performed using Microsoft Excel.

## Results

### Generation of *TRPV1::TRPV3* double knock-out mice

**Figure 1:**
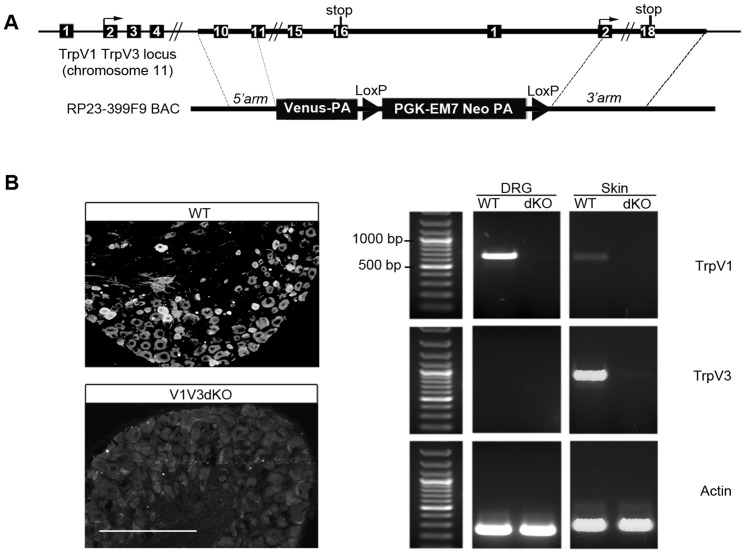
Generation and characterization of V1V3dKO mice. (A) Schematic structure of parts of *Trpv1 and Trpv3* genomic loci (top), and the targeting BAC construct showing the fusion of Venus cDNA to exon 11 of *Trpv1* gene followed by a loxP sites flanked selection marker *neo* (bottom). (B) Left panel: Immunohistochemistry analysis of DRG sections showing neurons expressing TRPV1 proteins in WT animals (top) but not in V1V3dKO mice (bottom). Right panel: RT-PCR analysis using total RNA extracted from DRG and skin of WT and V1V3dKO mice. *Trpv1* transcripts are detected after 35 cycles in the WT but not in V1V3dKO DRG samples. In these conditions we found low but detectable levels of *Trpv1* transcripts in skin samples. *Trpv3* transcripts are detected after 35 cycles in the WT but not in the V1V3dKO skin samples. Actin is used as a loading control.

Since *Trpv1* and *Trpv3* genes are within 10 kb of each other, we have designed a targeting construct using bacterial artificial chromosome (BAC-based) homologous recombination technology that allowed the replacement of an 18 kb genomic DNA fragment by a cassette containing the green fluorescent protein Venus and a floxed PGK-neomycin selection marker (Fig. 1A). The deleted region encompasses exons encoding transmembrane domains five and six, the C-terminus cytoplasmic tail, the 3′ untranslated region of *Trpv1*, the whole promoter region and the start codon-containing exon of *Trpv3*. Homozygous *Trpv1::Trpv3* double knock-out mice (hereafter V1V3dKO mice) were born in a Mendelian ratio, were fertile and presented the typical wavy hair characteristic of juvenile TRPV3-null mice.

**Figure 2:**
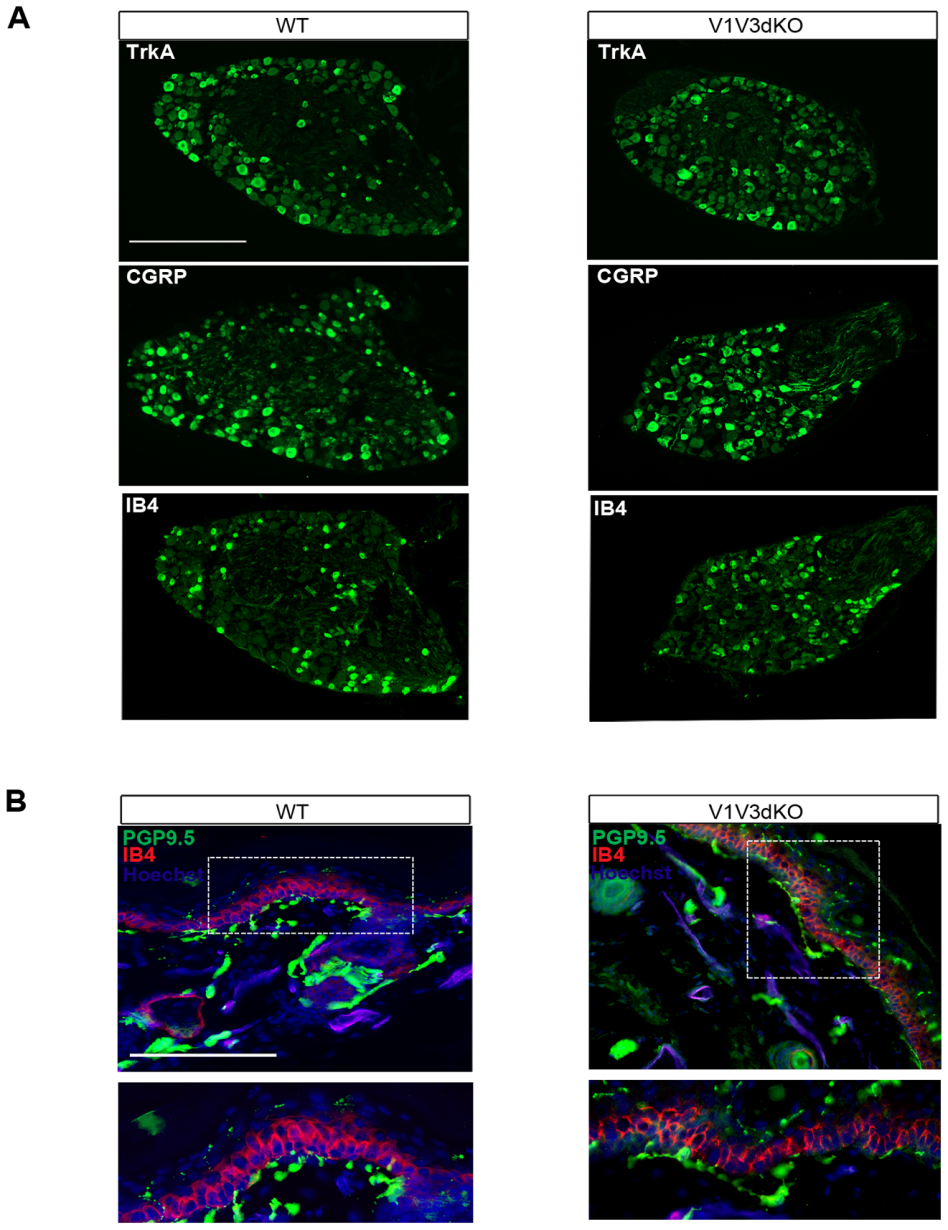
Expression of neuronal markers in V1V3dKO mice. (A) Immunohistochemistry analysis on DRG sections showing comparable expression of TrkA (top), CGRP (middle) and IB4 (bottom) in WT (left panels) and in V1V3dKO mice (right panels). Scale bar, 100 µm. (B) Double-staining of PGP 9.5 (green), Hoechst (blue) and IB4 (red) on glabrous skin sections revealing normal innervation of the paw in WT (left panel) and V1V3dKO mice (right panels). The two panels below show the magnification of each white rectangle.

We first verified that endogenous TRPV1 and TRPV3 expression was abolished in V1V3dKO mice. To test for loss of TRPV1 we used immuno-labeling experiment. Anti-TRPV1 antibody showed lack of staining in adult DRG neurons from V1V3dKO mice, whereas this same antibody stained a small subset of DRG neurons from wild-type mice (hereafter WT mice) (Fig. 1B, left panel). To test for loss of *Trpv3* transcripts, we performed reverse transcription polymerase chain reaction (RT-PCR). Using primers specific to *Trpv3*, we could amplify appropriate and high amount of cDNA from skin RNA samples of WT but not V1V3dKO mice (Fig. 1B, right panel). In these same conditions, very little amount of *Trpv1* cDNA could be amplified from WT but not from V1V3dKO skin RNA samples. Consistent with the predominant expression of TRPV3 in rodents skin [6], we could not amplify *Trpv3* from WT DRG RNA. In contrast, transcripts for F-actin were readily detected at normal levels in all tested tissues. To test whether loss of TRPV1 and TRPV3 channels perturbed the development and maturation of DRG neurons, we checked the expression of few markers of DRG neurons subsets. Qualitative analyses showed that both genotypes displayed normal expression of molecular markers of peptidergic and nonpeptidergic neuronal subsets, such as TrkA, CGRP and IB4 (Fig. 2A). Knowing the predominant expression of TRPV3 in the skin, we monitored the expression of markers of free nerve endings and skin keratinocytes. PGP9.5 staining revealed dense epidermal innervation and IB4 staining showed normal organization of skin keratinocytes in both genotypes (Fig. 2B). On note, there was no expression of the TRPV1-Venus fusion protein both in heterozygous and homozygous mice even after the excision of the floxed selection marker cassette (data not shown). Altogether, these data show that the targeting construct ensured efficient ablation of both TRPV1 and TRPV3 channels, and that ablation of both channels had no effect on either DRG neurons developmental maturation or the overall innervation of the skin. Our mouse model thus opens the first opportunity to analyze the *in vivo* functional consequence of concomitant TRPV1 and TRPV3 loss-of-function.

### TRPV1 and TRPV3 channels cooperate to accurately sense noxious heat stimuli

Appropriate analysis of V1V3dKO mice required fine comparison with single TRPV1 and TRPV3-KO mice. Because the available single TRPV1-KO and TRPV3-KO mice were in a pure C57BL/6J background, we performed the initial analyses of V1V3dKO mice in the C57BL/6J/129svJ mixed background (F2 mice) and confirmed the most significant findings in the C57BL/6J 9^th^ generation V1V3dKO mice (F9 mice). Unless otherwise mentioned, data shown in the main figures are from V1V3dKO F9 littermates.

**Figure 3:**
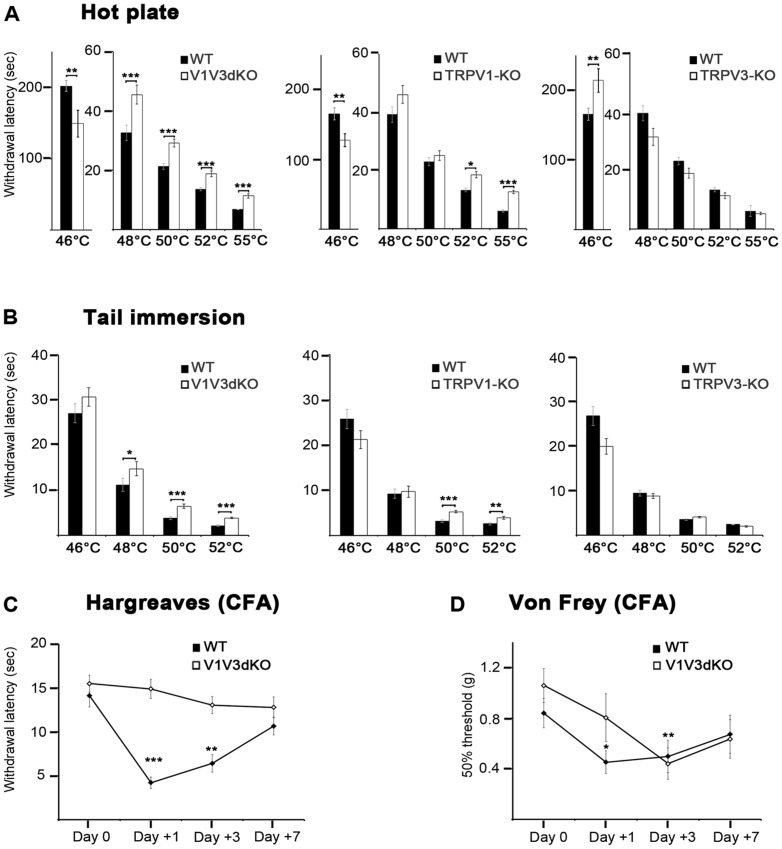
Impaired Behavioral Responses to Noxious Heat in V1V3dKO mice. (A) Latency to paw withdrawal in the hot plate test of WT and V1V3dKO mice (n = 15 WT, n = 12 knockouts) (left), WT and TRPV1-KO mice (n = 21 WT, n = 12 KO) (middle), WT and TRPV3-KO mice (n = 21 WT, n = 9 KO) (right). (B) Latency to tail withdrawal in the tail immersion test of WT and V1V3dKO mice (n = 15 WT, n = 12 KO) (left), WT and TRPV1-KO mice (n = 21 WT, n = 11 KO) (middle), and WT and TRPV3-KO mice (n = 21 WT, n = 9 KO) (right). (C) Latency to paw withdrawal of WT and V1V3dKO mice (n = 11 WT, n = 11 KO) in the Hargreaves test before (base line) and 24 hours, 3 days and 7 days after intraplantar injection of CFA. (D) 50% paw withdrawal threshold (g) of WT and V1V3dKO mice (n = 9 WT, n = 7 KO) in the Von Frey test using the up and down method, before (base line) and 24 hours, 3 days and 7 days after intraplantar injection of CFA. *p<0.05; **p<0.01; ***p<0.001. Data shown represent mean ± *sem*., CFA, Complete Freund's Adjuvant.

V1V3dKO mice appeared normal in body weight, open-field and activity profiles and cutaneous temperature, demonstrating that V1V3dKO mice do not have abnormalities in skin temperature, motor activity or anxiety (Fig. S1). To gain insights into the role of TRPV1 and TRPV3 channels in acute heat temperature sensation, we first subjected V1V3dKO and single TRPV1-KO and TRPV3-KO mice to classical thermosensory tests, including hot plate, tail immersion and thermotaxis gradient tests. When tested on a hot plate at four different noxious temperatures (48, 50, 52 and 55°C), V1V3dKO mice exhibited significant prolonged latency to paw licking or flicking at all tested temperatures. TRPV1-KO mice showed prolonged response latencies only at 52 and 55°C, whereas there was no phenotype in TRPV3-KO mice (Fig. 3A). Surprisingly, when tested at 46°C V1V3dKO and TRPV1-KO mice showed significant decreased response latencies whereas TRPV3-KO exhibited significantly prolonged response latency in comparison to their respective control mice. When tested in the tail immersion protocol, V1V3dKO mice exhibited prolonged response latencies at 48°, 50° and 52°C, TRPV1-KO mice had prolonged responses only at 50° and 52°C whereas no difference between control and TRPV3-KO mice could be observed (Fig. 3B). Of note, original studies using TRPV1-KO and TRPV3-KO mice in a C57BL/6J/129svJ mixed background showed very subtle or conflicting phenotypes in both experimental paradigms and did not describe the hot plate behavior of these mice at 46°C [11]–[15], [18], [19]. Together, the hot plate and the tail immersion data revealed two opposite phenotypes in V1VdKO mice: a reduced sensitivity in the noxious heat temperature range and intriguing hypersensitivity phenotype in the moderately noxious heat temperature range.

To address whether loss of TRPV1 and TRPV3 channels contributes to inflammatory pain, we inflamed the hind paw of WT and V1V3dKO mice using the complete Freund adjuvant (CFA), and evaluated their thermal and mechanical sensitivity before the inflammation, one day, three and seven days post-CFA (Figures 3C and 3D). For thermal sensitivity, we used the radiant heating assay and for mechanical sensitivity, we applied the Von Frey filaments using the up-down method [20]. In both paradigms baseline thermal and mechanical responses were similar between WT and V1V3dKO mice. One and three days post inflammation, WT mice exhibited a significant reduction in the withdrawal latencies that returned to baseline levels at seven days post-CFA. In contrast, V1V3dKO mice were totally resistant to CFA-induced thermal hyperalgesia as the withdrawal latencies did not change from baseline levels at any tested time points (Fig. 3C). This result is similar to that observed in TRPV1-KO mice [12], [15], and knowing that TPRV3-KO mice [11] developed pronounced CFA-induced thermal hyperalgesia, we concluded that V1V3dKO mice resistance to CFA-evoked thermal hyperalgesia is TRPV1 dependent. For the mechanical pain, using the same inflammatory agent, one day post-CFA WT mice exhibited significant mechanical hypersensitivity to Von Frey hair filaments that returned to almost baseline levels after seven days post-inflammation. V1V3dKO mice mechanical hypersensitivity reached significance only three days post-CFA, suggesting a slightly delayed CFA-induced mechanical pain (Fig. 3D).

**Figure 4:**
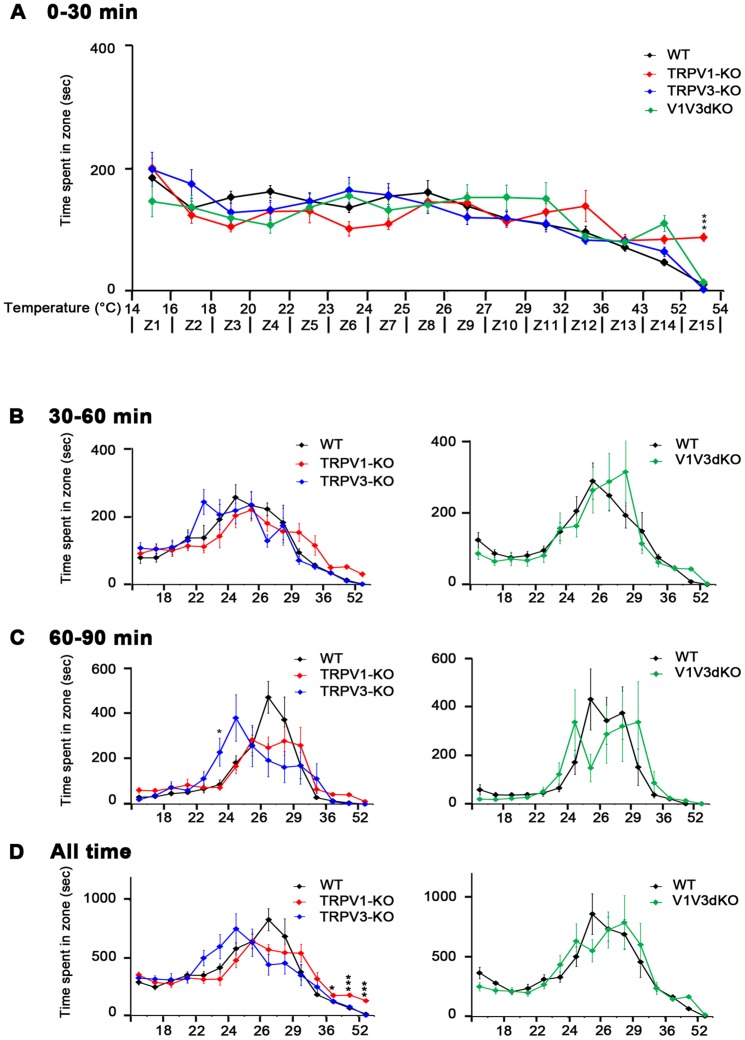
Behavior of TRPV1-KO, TRPV3-KO and V1V3dKO mice in the thermotaxis gradient assay. (A) Behavior of TRPV1-KO, TRPV3-KO and V1V3dKO mice in the temperature gradient assay during the first 30 minutes trial (n = 24 WT, n = 12 TRPV1-KO, n = 13 TRPV3-KO, n = 12 F9 V1V3dKO. All genotypes display normal exploratory behavior in the fifteen zones of the gradient (Z1-Z15) except TRPV1-KO that spend significant amount of time in the hottest area of the arena (Z14 and Z15). (B) Behavior of TRPV1-KO, TRPV3-KO and V1V3dKO mice in the temperature gradient assay during the second 30 minutes trial (n = 24 WT, n = 12 TRPV1-KO, n = 13 TRPV3-KO (left panel)), (n = 12 F9 V1V3dKO and n = 12 WT F9 littermates (right panel)). All three genotypes start showing a clear bias towards warm temperatures (23–28°C) in the middle of the arena (Z6–Z10). (C) Behavior of TRPV1-KO, TRPV3-KO and V1V3dKO mice in the temperature gradient assay during the last 30 minutes trial (n = 24 WT, n = 12 TRPV1-KO, n = 13 TRPV3-KO (left panel)),(n = 12 F9 V1V3dKO and n = 12 WT F9 littermates (right)). In comparison to WT mice, TRPV3-KO mice display a clear bias towards cooler temperatures with a peak of occupancy around 25°C, whereas TRPV1-KO mice occupy a wider temperature area (Z6 to Z12). V1V3dKO behave the same way as their control littermates. (D) Behavior of TRPV1-KO, TRPV3-KO and V1V3dKO mice in the temperature gradient assay during the whole trial (n = 24 WT, n = 12 TRPV1-KO, n = 13 TRPV3-KO (left panel)), (n = 12 F9 V1V3dKO and n = 12 WT F9 littermates (right panel)). TRPV1-KO mice show a slight bias towards hotter area of the arena (25° to 36°C) and TRPV3-KO mice have a tendency to occupy cooler parts of the arena (22° to 26°C). This bias phenotype of the single knock-outs seems to be attenuated in V1V3dKO mice.*p<0.05; **p<0.01; ***p<0.001 when comparing TRPV1-KO to WT mice. Data shown represent mean ± *sem*.

To further assess the contribution of TRPV1 and TRPV3 channels in acute thermosensation we subjected V1V3dKO mice and the individual TRPV1-KO and TRPV3-KO mice to the gradient assay. As previously described [11], in this thermotaxis assay, mice were allowed to move freely on a flat rectangular arena (10 cm by 125 cm) with the floor surface endowed with a temperature gradient ranging from 14° to 55°C along the length. The arena is virtually divided in 15 equidistant zones and the location of the mice in each zone is scored during a 90 minutes trial. The behaviors of the mice are presented in three consecutive thirty minutes-periods and during the whole trial. Of note, when the arena is set at room temperature, mice spent significantly more time at the corners of the arena in comparison to the center. Most importantly, mice spent almost equal amount of time at either corners, suggesting that no visual cues were interfering with the behavior of the mice (data not shown). When the floor temperatures are set from 14° to 55°C, all four genotypes displayed the prototypical thermotaxis behavior showing normal exploration of the whole arena during the first 30 minutes (Fig. 4A). TRPV1-KO showed an additional prolonged occupancy of the hottest area of the arena, further demonstrating the role of TRPV1 in sensing acute noxious heat temperature. In the second 30 minutes period, TRPV1-KO mice still displayed a clear bias towards hotter temperatures whereas TRPV3-KO mice started to show a shifted peak towards cooler temperatures. Comparison between WT and V1V3dKO mice showed no difference between the two genotypes (Fig. 4B). Importantly, during the last 30 minutes period, WT mice displayed a sharp occupancy of the arena around 27°C, TRPV1-KO mice occupancy was much wider than that of control mice (between 25,5°C and 32°C), whereas TRPV3-KO mice displayed a sharp bias towards cooler temperatures around 25°C (Fig. 4C). Interestingly, in all three 30 minutes' periods, V1V3dKO mice behaved the same way as their WT littermates (Fig. 4A–4C). When the behaviors of the 90 minutes trial were plotted, TRPV1-KO mice spent significantly higher amount of time in the 42°C to 55°C area of the arena and occupied a much wider temperatures zones (25°C to 36°C) compared to control mice whereas TRPV3-KO displayed an opposite behavior by occupying a wide cool temperature zones (22°C to 26°C) (Fig. 4D, left panel). Interestingly, behavioral biases displayed by the single knock-out mice were drastically abolished in V1V3dKO mice (Fig.4D, right panel), further suggesting a functional interaction between TRPV1 and TRPV3 channels in acute temperature sensation.

### TRPV1 and TRPV3 channels are required for proper perception of moderate noxious heat temperature

**Figure 5:**
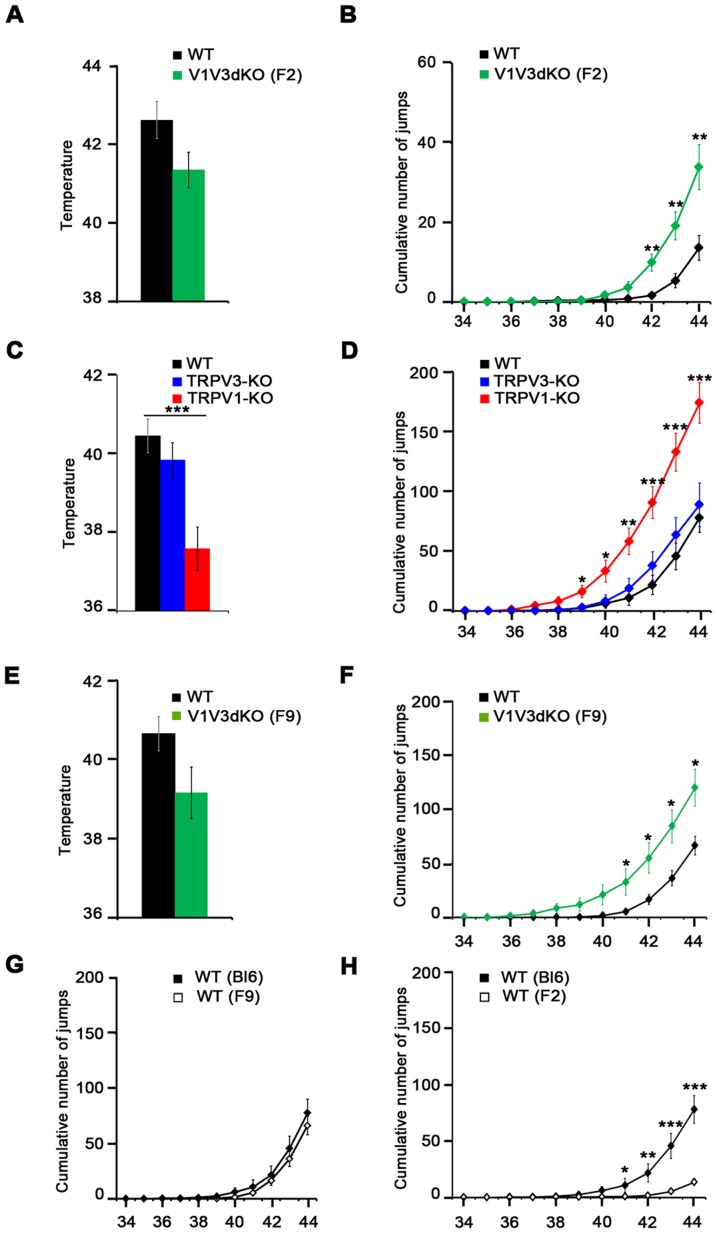
Behavior of TRPV1-KO, TRPV3-KO and V1V3dKO mice in the dynamic hot plate assay. Responses of the mice to a 10 minutes dynamic hot plate assay (DHP) with the floor temperature increasing from 34° to 44°C at a rate of 1° per minute. (A) Latency to the first jump of WT and V1V3dKO mice in the C57BL/6J/129svJ mixed background (n = 22 WT, n = 24 V1V3dKO). (B) Cumulative number of jumps of WT and V1V3dKO mice in the C57BL/6J/129svJ mixed background (n = 22 WT, n = 24 V1V3dKO). As compared to WT, the V1V3dKO mice show a pronounced escape behavior. (C) Latency to the first jump of WT, TRPV1-KO and TRPV3-KO mice (n = 18 WT, n = 12 TRPV1-KO, n = 9 TRPV3-KO). (D) Cumulative number of jumps of WT, TRPV1-KO and TRPV3-KO mice (n = 18 WT, n = 12 TRPV1-KO, n = 9 TRPV3-KO). The behaviors of the three genotypes plotted together highlight the particularly significant increase of the escape behavior in TRPV1-KO mice. (E) Latency to the first jump of F9 WT and V1V3dKO F9-littermates (n = 15 WT, n = 12 V1V3dKO). (F) Cumulative number of jumps of F9 WT and V1V3dKO F9-littermates (n = 15 WT, n = 12 V1V3dKO) in the DHP test. (G) Comparison of the DHP behavior between F9 WT and C57BL/6J mice shows no difference between the two control mice (n = 18 C57BL/6J, n = 15 WT F9). (H) Comparison of the DHP behavior between F2 WT and C57BL/6J mice shows a 6-fold difference in the total number of jumps (n = 20 F2 WT, n = 18 C57BL/6J). *p<0.05; **p<0.01; ***p<0.001. Data shown represent mean ± *sem*. In all panels, WT animals are represented in black square, TRPV1-KO in red, TRPV3-KO in blue and V1V3dKO in green.

Phenotypic characterization of acute heat temperature sensation in previously described thermoTRPV null mice used the classical nociception tests like hot plate, tail immersion and thermotaxis assays [11], [12], [15], [18]. To further characterize the role of TRPV1 and TRPV3 channels in acute thermosensation, we tested the behavior of all three genotypes in the recently described dynamic hot plate (DHP) paradigm [21]. In this test, mice are allowed to move freely in an 11.5 cm by 11.5 cm by 20 cm chamber with the floor temperature linearly increasing from 34° to 44°C at a rate of one degree per minute. Multiple parameters such as latency to first jump, number of jumps every minute and cumulative number of jumps during the whole trial can be measured. Figure 5 shows the behavior of F2 V1V3dKO and their WT littermates. We found no significant difference in the latency to the first jump between the two genotypes (Fig. 5A). Surprisingly, V1V3dKO mice exhibited a 2.4 fold increase in the number of jumps (13.6±3 for WTs and 33.6±5.6 for V1V3dKO mice n = 20 and 23 respectively) (Fig. 5B).

Is this enhanced escape behavior phenotype specific to ablation of TRPV1 or TRPV3 alone, or due to absence of both channels? To answer this question we subjected WT C57BL/6J and single TRPV1-KO and TRPV3-KO mice to this same experimental protocol. Surprisingly, TRPV1-KO mice exhibited a drastic decreased latency to the first jump (37.58±0.54 for TRPV1-KO mice versus 40.43±0.42 for WTs, n = 12 and n = 18 respectively) (Fig. 5C) and exhibited a 2.28 fold increase in the total number of jumps (173.9±16.9 jumps for TRPV1-KO versus 76.11±8.51 for WTs) (Fig. 5D). TRPV3-KO mice displayed an almost WT-like behavior (Fig. 5C and 5D, blue traces), suggesting that the observed enhanced escape behavior in F2 V1V3dKO mice is likely due to TRPV1 loss-of-function with little or no contribution of skin-derived TRPV3 channel.

Knowing the influence of the mice background on behavioral outcomes and in order to properly compare the behavior of single TRPV1-KO and TRPV3-KO mice to that of V1V3dKO mice, we monitored the performance of F9 V1V3dKO and their WT littermates in the DHP paradigm. In this background, we found no significant difference in the latency to the first jump (Fig. 5E). However, in this background, V1V3dKO mice displayed a slightly significant higher number of jumps in comparison to their WT littermates (119.75±16.86 jumps for V1V3dKO versus 66.33±8.5 for WTs) (Fig. 5F), suggesting that loss of TRPV3 in TRPV1-KO background significantly decreased the massive escape behavior observed in TRPV1-KO mice. This hypothesis is supported by the fact that we found no difference in the total number of jumps between pure C57BL/6J and F9 WT littermates of V1V3dKO mice (Fig. 5G), demonstrating that the data obtained with the single TRPV1 and TRPV3-KO mice can be accurately compared to data obtained with F9 V1V3dKO mice. To further highlight the importance of the mice backgrounds, we found a 6 fold difference in the number of jumps between WT F2 and C57BL/6J mice (Fig. 5H). Taken together, the hot plate, the tail immersion, the thermotaxis gradient and the DHP results demonstrate that TRPV1 and TRPV3 channels cooperate with each other to modulate acute heat temperature sensation *in vivo*. Furthermore, the DHP paradigm revealed an intriguing and unexpected enhanced escape behavior in TRPV1-KO mice that seemed to be attenuated by the concomitant loss of TRPV3 channel.

## Discussion

The chromosomal location of *Trpv1* and *Trpv3* genes greatly impeded the generation of TRPV1::TRPV3 double knock mice by simply crossing previously generated single knock-outs [11], [12], [15]. To circumvent this obstacle, we decided to generate such a mouse model using BAC-based homologous recombination in embryonic stem cells. V1V3dKO animals displayed additive deficits in a well-defined window of temperature in a series of thermally evoked pain behaviors such as the hot plate, the tail immersion and the tail flick assay. In the gradient assay, the temperature bias displayed by single TRPV1-KO and TRPV3-KO mice was significantly attenuated in V1V3dKO mice and the remarkable and intriguing TRPV1-dependent enhanced escape behavior in the dynamic hot plate paradigm was also attenuated in V1V3dKO. These findings are in agreement with the well-described temperature activation overlap between TRPV1 and TRPV3 channels (42°C to 50°C) [1], [6] and provide *in vivo* evidence for a cooperative role between skin-derived TRPV3 and primary sensory neurons-enriched TRPV1 channels in controlling acute heat temperature sensation. Our results also revealed a new and previously unidentified TRPV1-dependent hypersensitivity phenotype and demonstrate that heat temperature sensation requires other molecules in addition to thermo-TRPV channels.

Two recent studies from Caterina's laboratory investigated the extent to which TRPV2, TRPV3 and TRPV4 channels could contribute to the residual heat sensitivity encountered in TRPV1-KOmice [13], [18]. Analysis of TRPV1::TRPV2 double knock-out mice or desensitization of TRPV1 afferents using resiniferatoxin (RTX) revealed no additive thermosensory defects, providing a clear demonstration that TRPV2 channel is dispensable for temperature sensation *in vivo* [13]. Masking TRPV1 channel's activity by the TRPV1 antagonist JNJ-17203212 in TRPV3::TRPV4 double knock-out mice also revealed no additive defects in a series of acute heat temperature tests, suggesting that TRPV1 is the master thermoTRPV channel required for acute heat temperature sensation *in vivo* [18]. Whether pharmacological inhibition of TRPV1 activity in TRPV3::TRPV4 double knock-out mice fully recapitulates TRPV1 loss-of-function remains an open question. Furthermore, the hyperthermic effects of TRPV1 antagonists [22], [23] likely interfere with the behavioral readouts in most of the thermosensory tests applied to these animals.

Here, we showed that when subjected to the hot plate assay, V1V3dKO mice exhibited increased response latencies at 48°C and 50°C. At these same temperatures, single TRPV1-KO and TRPV3-KO mice behaved the same as their WT littermates. V1V3dKO mice displayed the same response profile in the tail immersion protocol, demonstrating that skin-derived TRPV3 and primary sensory neurons-enriched TRPV1 are required for sensing and transducing a well-defined window of moderately noxious heat temperature *in vivo*. The functional interaction between TRPV1 and TRPV3 channels in acute heat temperature sensation was further demonstrated in the gradient test. In the gradient assay, TRPV1-KO and TRPV3-KO mice displayed opposite behaviors. TRPV1-KO mice showed a tendency to explore hotter areas of the arena whereas TRPV3-KO mice exhibited a clear bias towards cooler temperatures. Interestingly, this temperature bias of both single knock-outs was completely abolished in V1V3dKO mice. Huang and colleagues [18] extensively explored the cooperative role between TRPV3 and TRPV4 channels in the gradient test paradigm. They showed that TRPV3-KO mice on the 129S6 background exhibited a clear bias towards cooler floor temperatures whereas the same mice on the C57BL6 background behaved like WT mice, contradicting results described in our original study [11] in which we showed a drastic difference between WT and TRPV3-KO mice on the 129S6/C57BL6 mixed background. In this study, we used the same TRPV3-KO mice that have been analyzed in Huang et al. study and showed that TRPV3-KO mice exhibited a clear bias towards cooler temperatures, confirming the results of Moqrich et al study [11]. What can explain the discrepancies between our study and the one described in Huang and colleagues, knowing that the same mice have been used in both studies? We favor the hypothesis that the observed differences are due the different parameters used in the gradient assay. In Huang et al. study, the temperatures at the edges of the arena are set between 0.8° and 48.8°C, whereas in our study the temperature edges are set between 14° and 55°C. In the first set up, the gradient is sharp enough to push the mice away from the corners, whereas in our set up the gradient is loose enough to allow more exploration of the cooler side of the arena, thus opening a much larger window to identify the subtle impaired thermosensory phenotype in TRPV3-KO mice. In our set up, we also succeeded to identify two distinct phenotypes in TRPV1-KO mice: the increased time spent at the noxious heat area of the arena and a biased occupancy towards the warmer side of the arena. Interestingly, the opposite temperature biases displayed by single knock-out mice is totally abolished in V1V3dKO mice, further demonstrating the functional interaction between TRPV1 and TPRV3 channels.

Using classical thermosensory tests such as the hot plate, the tail immersion, the temperature gradient and the Hargreaves tests we confirmed that TRPV1-KO mice exhibited prolonged withdrawal latencies in response to acute noxious heat temperatures as well as a drastic resistance to CFA-induced thermal hyperalgesia. However, using the DHP test, we found that TRPV1-KO mice exhibited an intriguing and extraordinary enhanced escape behavior, revealing a previously unidentified phenotype in TRPV1-KO mice. How loss of TRPV1 channel could lead to such hypersensitivity in response to increasing heat temperature in the DHP test?

TRPV1 channel has been envisioned as the most promising channel for pain therapy. Because of the undesirable hyperthermia effects of TRPV1 antagonists [22], extensive works deciphering the general tissue distribution and the role of TRPV1 in thermoregulation have been launched [23]. TRPV1 channels are highly enriched in primary sensory neurons whose afferents innervate the skin and visceral organs, including the gastrointestinal tract, large intestine and urinary bladder [24]–[26]. However, TRPV1 expression in the central nervous system (CNS) is still under intense debate. A recent study, allowing genetic tracing of TRPV1-expressing cells confirmed the high expression of TRPV1 in peripheral sensory neurons and revealed some expression in discrete regions of the brain namely in the caudal hypothalamus [24], [27], [28], suggesting that the functional role of TRPV1 is mainly mediated through its activity outside the brain. Whether TRPV1 in peripheral primary sensory neurons play a role in thermoregulation is still controversial. Indeed, TRPV1-KO mice exhibited no changes in body temperature in response to increased or decreased ambient temperature [29]–[32], suggesting that TRPV1 is dispensable for thermoregulation *in vivo*. In contrast, peripherally restricted TRPV1 antagonists caused pronounced hyperthermia both in rats and mice [33]–[35]. TRPV1 antagonism-induced hyperthermia is abolished in TRPV1-KO mice, suggesting that tonically active peripheral TRPV1 plays a predominant role in thermoregulation [33]–[35]. Furthermore, desensitization of visceral TRPV1 using low doses of RTX abolished peripherally restricted antagonist-mediated hyperthermia, demonstrating that visceral TRPV1 channels are likely to regulate body temperature [33]. Behavioral discrepancies between TRPV1 antagonism and TRPV1 loss-of-function might be explained by compensatory mechanisms in the mutant mice. In our study, we showed that challenging TRPV1-KO mice in a behavioral paradigm in which the floor temperature is linearly increasing from 34° to 44°C provoked two distinct behaviors that can be qualified as a state of hypersensitivity: a decreased latency to trigger the first jump during the ramp protocol and a significantly higher number of jumps at the end of the protocol. Whether this hypersensitivity phenotype is due to a state of hyperthermia is difficult to measure under our experimental conditions, but deeper analysis of the mechanisms that induce this hypersensitivity-like phenotype in TRPV1-KO mice is warranted. An important observation that came out from our DHP paradigm is that the latency to trigger the first jump and the total number of jumps were significantly reduced in V1V3dKO, further suggesting a functional interaction between TRPV1 and TRPV3 channels and may be providing hints into how skin-derived TRPV3 and primary sensory neurons-enriched TRPV1 cooperate with each other to allow proper perception of acute heat temperature.

In vitro studies showed that TRPV1 and TRPV3 channels have an overlapping temperature activation window that lies within the moderate heat noxious range (42°C to 55°C). TRPV3 activation threshold is around 33°C and its activity increases with increasing temperatures as well as following repeated heat stimulations [6], [7], [11]. TRPV3 has been recently shown to be critical for ATP and nitric oxide (NO) production in keratinocytes upon heat stimulation [36], [37] and NO donors are potent and direct activators of TRPV1 [37]. Moreover, mice overexpressing TRPV3 in keratinocytes produced more prostaglandin E2 in response to heat. Importantly, pharmacological inhibition of TRPV1 with JNJ-17203212 in TRPV3 overexpressing mice led to increased escape responses to noxious heat [38]. Based on these data, we propose a hypothesis in which, in the DHP conditions, increasing heat application to the skin will activate and sensitize TRPV3 activity in keratinocytes. Activated keratinocytes will in turn release a series of bioactive substances such as ATP, NO, PGE2 and other algogenic compounds, some of which will lead to heat-independent activation of peripheral TRPV1 channel. TRPV1 activation will regulate neuronal activity as well as the vascular tone, thus keeping core body temperature at its normal level [23], [33], [39]. In mice where TRPV1 activity is blocked through pharmacological or genetic means, TRPV1-dependent body temperature control is impaired and hyperthermia occurs. Alternatively, the escape behavior observed in TRPV1-KO mice might simply be related to an elevated activity due to deficient heat temperature sensation in these mice. In line with this hypothesis, previous studies have shown that loss of TRPV1 channels leads to hyperactivity in juvenile mice [32] and to decreased anxiety-related behavior [40]. Analyses of TRPV1 conditional knock-out mice in which TRPV1 is inactivated in peripheral or central neurons will provide valuable insights into the role of TRPV1 in heat-induced versus stress- or hyperactivity-induced escape behavior.

## Supporting Information

Figure S1 General phenotype of the V1V3dKO mice. (A) Growth profiles of WT mice and V1V3dKO (n = 7 WT and n = 6 F2 V1V3dKO). (B) Open field exploratory behavior. The results show the time spent in the corner and the center of the arena during the 5 minutes trial. No difference between WT and V1V3dKO mice was revealed using this paradigm. (C) Locomotor activity of the V1V3dKO mice during the thermotaxis gradient assay. This spontaneous locomotor activity was monitored in the gradient apparatus with no temperature during 90 minutes (n = 9 WT littermates and n = 9 F2 V1V3dKO). The total distance covered (in meters) and the resting time of the mice (in minutes) were recorded. *p<0.05; **p<0.01; ***p<0.001. Data shown represent mean ± *sem*. (D) Cutaneous temperature of the V1V3dKO mice. Local cutaneous temperature on the hind limb was measured using a surface type-T thermocouple probe placed on a hairless skin area. The thermocouple was connected to an electronic thermometer (BAT-12; Physitemp Instruments Inc, Clifton, NJ, USA). No difference in cutaneous temperature was detected between WT and V1V3dKO mice. *p<0.05; **p<0.01; ***p<0.001. Data shown represent mean ± *sem*.
(TIF)
