# Supplementary figures and images for: Acute Heat-Evoked Temperature Sensation Is Impaired but Not Abolished in Mice Lacking TRPV1 and TRPV3 Channels
Source: PLoS One. 2014 Jun 12;9(6):e99828. doi: 10.1371/journal.pone.0099828 (PMC4055713; doi:10.1371/journal.pone.0099828)

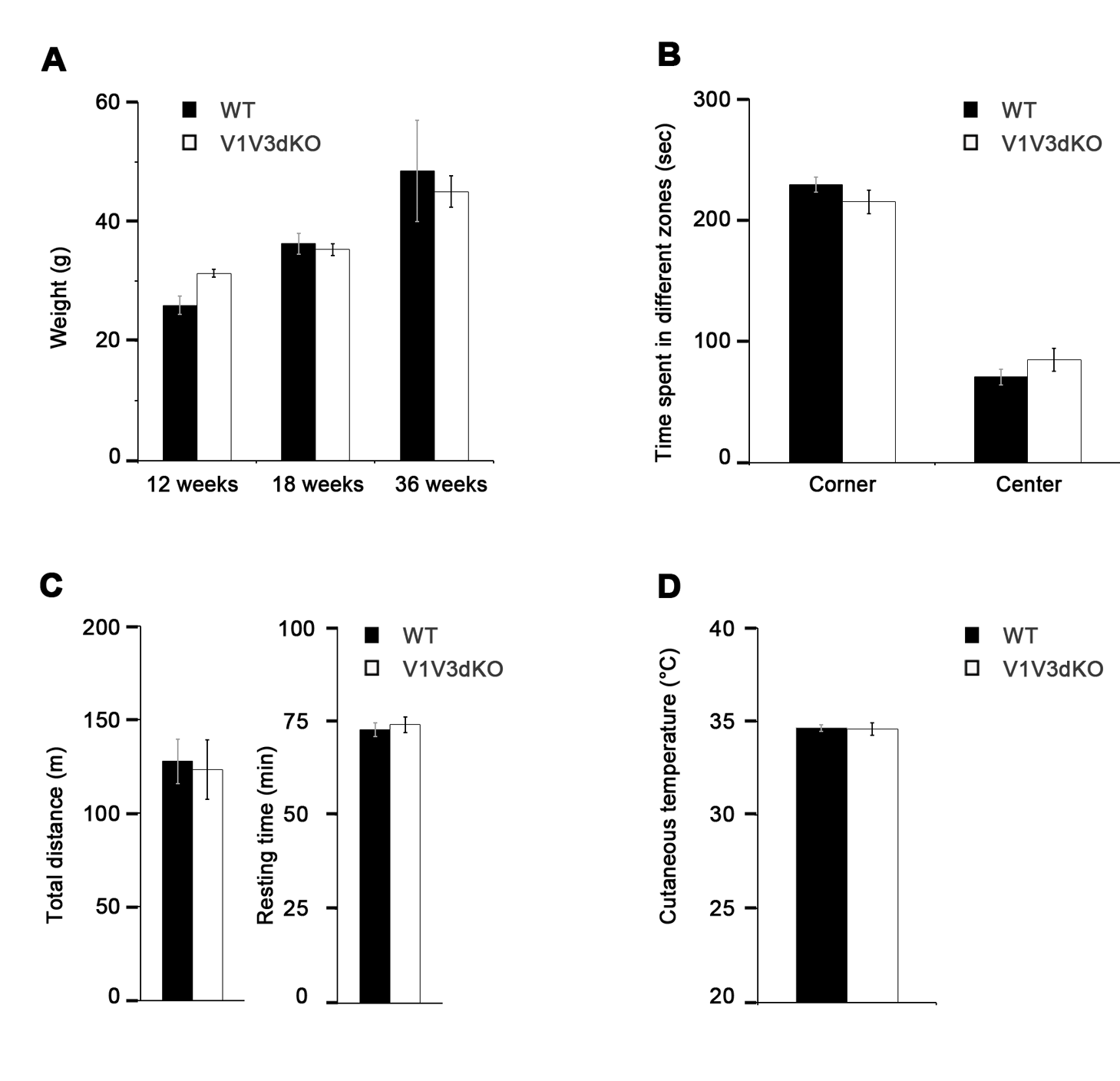

Supplement: Figure S1 — General phenotype of the V1V3dKO mice. (A) Growth profiles of WT mice and V1V3dKO (n = 7 WT and n = 6 F2 V1V3dKO). (B) Open field exploratory behavior. The results show the time spent in the corner and the center of the arena during the 5 minutes trial. No difference between WT and V1V3dKO mice was revealed using this paradigm. (C) Locomotor activity of the V1V3dKO mice during the thermotaxis gradient assay. This spontaneous locomotor activity was monitored in the gradient apparatus with no temperature during 90 minutes (n = 9 WT littermates and n = 9 F2 V1V3dKO). The total distance covered (in meters) and the resting time of the mice (in minutes) were recorded. *p<0.05; **p<0.01; ***p<0.001. Data shown represent mean ± sem. (D) Cutaneous temperature of the V1V3dKO mice. Local cutaneous temperature on the hind limb was measured using a surface type-T thermocouple probe placed on a hairless skin area. The thermocouple was connected to an electronic thermometer (BAT-12; Physitemp Instruments Inc, Clifton, NJ, USA). No difference in cutaneous temperature was detected between WT and V1V3dKO mice. *p<0.05; **p<0.01; ***p<0.001. Data shown represent mean ± sem. (TIF) [file pone.0099828.s001.tif]
